# Proanthocyanidins Inhibit Osteoblast Apoptosis via the PI3K/AKT/Bcl-xL Pathway in the Treatment of Steroid-Induced Osteonecrosis of the Femoral Head in Rats

**DOI:** 10.3390/nu15081936

**Published:** 2023-04-18

**Authors:** Hui Li, Yufei Zhang, Yangquan Hao, Peng Xu, Xingyu Wang, Bin Zhu, Chao Lu, Ke Xu

**Affiliations:** 1 Department of Joint Surgery, Xi’an Hong Hui Hospital, Xi’an Jiaotong University Health Science Center, Xi’an 710054, China; 2 Department of Traditional Chinese and Western Medicine, First Clinical School of Shaanxi University of Traditional Chinese Medicine, Xianyang 712046, China; 3 College of Food Engineering and Nutritional Science, Shaanxi Normal University, Xi’an 710062, China; 4 Department of Pharmacy, Beijing Tiantan Hospital, Capital Medical University, Beijing 100000, China

**Keywords:** SONFH, proanthocyanidins, network pharmacology, apoptosis, PI3K/AKT signaling pathway

## Abstract

Background: Steroid-induced osteonecrosis of the femoral head (SONFH) is a common clinical disease caused by massive or prolonged use of steroids. Its pathogenesis is unclear, but its incidence is increasing annually. It is characterized by an insidious and rapid onset, and high disability rate, causing a great burden on patients’ daily life. Therefore, clarifying its pathogenesis and providing early and effective treatment for steroid osteonecrosis is important. Methods: In vivo, we used methylprednisolone (MPS) to construct a SONFH rat model and employed Mirco-ct, Hematoxylin and eosin (H&E) staining, and TdT-mediated dUTP nick end labeling (TUNEL) staining analysis to evaluate the therapeutic effects of proanthocyanidins (PACs). Network pharmacology analysis was conducted to mine targets associated with femoral head necrosis, and PACs analyzed possible molecular mechanisms. In vitro, PACs were added at different doses after treatment of cells with dexamethasone (DEX), and human osteoblast-like sarcoma(MG-63) cell apoptosis was determined by Annexin V-FITC-PI. The mechanisms by which PACs regulate bone metabolism via the Phosphoinositide 3-kinase(PI3K)/protein kinase B(AKT)/Recombinant Human B-Cell Leukemia/Lymphoma 2 XL(Bcl-xL) axis were explored by Western blotting. Result: In vivo studies showed that PACs prevented SONFH in rat model. The PI3K/AKT/Bcl-xL signaling pathway was selected by network pharmacology approach; in vitro studies showed that proanthocyanidin-activated AKT and Bcl-xL inhibited osteoblast apoptosis. Conclusions: PACs can inhibit excessive osteoblast apoptosis in SONFH via the PI3K/AKT/Bcl-xL signaling axis and have potential therapeutic effects.

## 1. Introduction

Clinically, prolonged or localized and short-term usage of steroids has been widely applied in the treatment of immune diseases, which may increase the risk of steroid-induced osteonecrosis of the femoral head (SONFH) [1,2]. Recent epidemiological studies have found an increase in the proportion of SONFH [3,4]. Patients with end-stage femoral head necrosis are usually treated with total hip arthroplasty; however, some younger patients often require one or more revision surgeries owing to the limited lifespan of the prosthesis [5]. Therefore, it is crucial to identify an appropriate treatment for early femoral head necrosis and delay the time to arthroplasty.

It has been found that proanthocyanidins (PACs) can play a protective role in a variety of diseases through its antioxidant, anti-inflammatory, anti-apoptotic properties, among various others [6,7,8]. Wu et al. studied the effect of proanthocyanidin B2 on the most common malignant primary bone tumor in orthopedics and proved that proanthocyanidin B2 could suppress and promote osteosarcoma (OS) cell proliferation and apoptosis, respectively [9]. Previous studies reported that PACs inhibit oxidative damage and apoptosis, thereby reducing the incidence of SONFH in rabbit models. They demonstrated that PACs could markedly attenuate the incidence of SONFH through its antioxidant and anti-apoptotic properties [10,11]. Sun et al. screened out differentially expressed genes through the Gene Expression Omnibus (GEO) database and investigated their roles with siRNA-Forkhead Box O1(FOXO1) and insulin-like growth factors −1 (IGF-1)(Phosphoinositide 3-kinase((PI3K)/protein kinase B (AKT) agonist) [12]. Although they drew a favorable conclusion, it remains uncertain whether there are any additional signaling pathways implicated.

To further reveal the relationship between the disease mechanism of femoral head necrosis and the action of PACs, the network pharmacology approach proposed by Andrew L. Hopkins at Dundee University, UK, in 2007 was used [11,13]. Drug efficacy, toxicity, and metabolic properties have been revealed in systems biology, biological network construction, and analysis, making full use of knowledge related to relevant targets, drugs, disease biomarkers, or pathways, and performing biocomputation to elucidate unknown mechanisms.

In SONFH, osteoblasts play a vital role in promoting bone formation to maintain bone homeostasis. Steroids bind to the steroid receptors present on the osteoclasts, causing a change in cell conformation, which in turn leads to the translocation or inhibition of the corresponding transcription factors, resulting in excessive apoptosis of osteoblasts and further development of osteonecrosis [14,15]. Therefore, implementing different ways to reduce excessive apoptosis of osteoblasts is one of the directions for treating SONFH [14,16].

We verified that PACs protected rats from bone loss due to PACs using animal models. The effect of PACs on osteoblast apoptosis and the related mechanisms of action were investigated in vitro by determining the pathways associated with femoral head necrosis using network pharmacology techniques. We investigated whether PACs could be used for the treatment of early femoral head necrosis.

## 2. Materials and Methods

### 2.1. Mice, Reagents, and Antibodies

A total of 45 six-week mature female Sprague–Dawley (SD) rats, weighing 190–220 g, were obtained from the Animal Center of Xi’an Jiaotong University (Xi’an, China) and maintained under a 12-h light/dark cycle at 22–24 °C and 55–60% humidity until 12 weeks of maturity, according to the guidelines of the Institutional Animal Care and Use Committee (Approval number SUCMDL20221128002). The human osteoblast-like sarcoma (MG-63) cell line was obtained from the Cell Bank of the Chinese Academy of Sciences (Shanghai, China). To assess the effect of PACs on dexamethasone (DEX)-treated cells, the cells were exposed to 100 μM DEX(Sigma-Aldrich, Milwaukee, WI, USA) and different concentrations of PACs (Macklin, Shanghai, China).

### 2.2. Rat Model Establishment and Treatment

Forty-five 12-week mature female SD rats, weighing 280–330 g, were randomly assigned to control (*n* = 15), steroid (*n* = 15), and PACs (*n* = 15) groups using the number table method. The control group was housed normally to establish the baseline of the batch; the steroid group was injected with lipopolysaccharide (LPS) 10 μg/kg (Sigma-Aldrich, USA) via tail vein at 24 h intervals on two consecutive days, and then injected with methylprednisolone (MPS) 40 mg/kg in the gluteal muscle (alternately, such as first on the left side and second on the right side) at 24 h interval for one week, for a total of three times, and 80,000 U of penicillin sodium was injected intraperitoneally twice a week to prevent infections. The PACs group was treated in the same way as the steroid group, and PACs was given by gavage at a daily dose of 20 mg/kg after the first injection of MPS for six weeks.

### 2.3. MicroCT to Detect Bone Density and Bone Microstructure of the Femur

Rats were euthanized after six weeks, and the structurally intact femoral heads from control, steroid-treated, and PACs-treated rats were preserved in 10% paraformaldehyde for 24 h. Micro-CT (SKYSCAN 1276; Bruker AXS GmbH, Karlsruhe, Germany) was used to assess bone density, bone mass, and trabecular microarchitecture at the femoral head site. Sample parameters calculated from these data included bone trabecular density (trabecular BMD), trabecular number (Tb·n, 1/mm), trabecular thickness (Tb·Th, mm), trabecular separation (Tb·Sp, mm), and bone volume-to-tissue volume ratio (BV/TV, %). Scanning parameters were as follows: current 400 μA, voltage 60 kV, 360-degree rotational scanning with an angular gain of 2 degrees, and scanning time of approximately 20–30 min per specimen. The entire femur was scanned to reconstruct a three-dimensional image with a resolution of 12 μm, and the selected area in the distal femoral epiphysis was scanned for at least 80 layers. These planar images were integrated into 3D images using the MicroView V.2.1 software.

### 2.4. Hematoxylin and Eosin (H&E) and TdT-Mediated dUTP Nick End Labeling (TUNEL) Staining

Femur samples were fixed in 4% paraformaldehyde for one day, decalcified in 12% Ethylene Diamine Tetraacetic Acid (EDTA) for two weeks, and then embedded in paraffin. The samples were sliced at 5 μm thickness, dewaxed in xylene, rehydrated through a series of graded ethanol, and then rinsed in distilled water. Representative images of histological observations were obtained using a light microscope after H&E staining. The percentage of empty lacunae was evaluated according to the previous method [17].

Apoptosis was detected and quantified at the cellular level using a TUNEL kit based on labelling of DNA strand breaks. Briefly, reagents were added dropwise after the repair of the broken membrane. Nuclei were re-stained using 4′,6-diamidino-2-phenylindole (DAPI) followed by incubation for 10 min in a light-proof environment, and slices were sealed using an anti-fluorescence burst sealer. Each section was photographed by randomly selecting at least three tissue-filled fields with consistent background light. Subsequently, we used Image-Pro Plus 6.0 software to determine the total cells with identically labelled DAPI blue nuclei and positive cells with identically labelled green fluorescent nuclei. We counted the number of total and positive cells in each image and calculated the apoptosis rate (%), (percentage of positive cells = positive cells (number of positive cells/total cells × 100%).

Empty bone traps caused by osteonecrosis have some similarity to those caused by artifacts from the decalcification process, but decalcification-induced empty bone traps result in wrinkling of the cell wall without significant changes in the nucleus. Moreover, we used the EDTA decalcification method, which preserves the morphological structure of bone tissue better [18]. The staining results revealed loss of continuity of trabeculae in SONFH, visible microfractures, disorganized arrangement, no cells seen in the trabecular fossa, degenerative necrosis of bone marrow cavity cells, and massive connective tissue proliferation, whereas the normal femoral head site had intact trabecular structure and no significant changes in bone cells, which were used to differentiate the calculations.

### 2.5. Network Pharmacology Analysis

The structure of PACs was obtained using the PubChem database, and targets with a Norm Fit prediction score > 0.7 were imported into the Swiss Target Prediction, PharmMapper, and Similarity ensemble approach (SEA) databases to acquire the relevant targets. The target identities were corrected and de-duplicated using the Universal Protein (UniProt) database [19]. The Online Mendelian Inheritance in Man (OMIM) and Genecards databases were searched with the keywords “Steroid-induced osteonecrosis of the femoral head” and “femur head steroid necrosis” to obtain the disease targets [20]. The drug and disease targets were entered into the Venny2.1 online software mapping tool platform, Venn diagrams were constructed, and the drug-disease common targets were retrieved by taking the intersection of the two. PACs and drug-disease common targets were entered into Cytoscape software to create a “disease-target-component” network diagram [21]. The following steps were used for establishing the Protein–Protein Interaction (PPI) network: Input the above drug-disease common targets into the Search Tool for the Retrieval of Interaction Gene/Proteins (STRING) database; set the protein type and minimum interaction threshold as “Homo sapiens” and 0.4, respectively; remove the isolated nodes; and construct the PPI network of protein interactions [22]. The PPI network was visualized by Cytoscape 3.9.2, and topological analysis was performed using the Network Analyzer tool. Genes with degree values above the mean score were selected as core targets by degree sorting, and the targets were plotted on a bar graph using R 4.2.1. Gene Ontology (GO) and Kyoto Eecyclopedia of Genes and Genomes (KEGG) enrichment analyses were performed on common targets using the Database for Annotation, Visualization and Integrated Discovery (David) [23]; the enrichment results were visualized and bar graph bubble plots were drawn. The Standard Delay Format (SDF)-format PACs were imported into ChemDraw 3D, and the Material Management (MM2) module was used to minimize energy and obtain the lowest energy advantage concept. Relevant protein structures were downloaded from the UniProt database and visualized separately using PyMOL. Autodock vina 1.1.2 was employed to dock ligands and receptors, and the conformation with high scores was selected for visual analysis of the interaction.

### 2.6. Cell Culture and Grouping

#### 2.6.1. Cell Culture

MG63 cells were cultured in Dulbecco’s modified Eagle medium (DMEM) containing 5.6 mmol/L glucose, 10% Fatal bovine serun (FBS), 100 U/mL penicillin, and 100 U/mL streptomycin, and then maintained at 37 °C with 5% CO_2_ and saturated humidity. MG63 cells were sub-cultured every other day and allowed to grow up to 80–90% fusion for passaging (48–72 h). Cells were passaged as follows: original culture medium was discarded and the cells were rinsed with 2 mL of phosphate buffer saline (PBS) solution. Trypsin (1 mL) was added to the cells using a pipette to digest the cells, followed by incubation for 30–60 s. The degree of digestion, when the cell edges started to become round, was observed, the culture flask was kept upright, trypsin was then discarded, and culture medium was quickly added to the cells using a pipette to terminate the effect of trypsin. Then, the cells were detached gently using a pipette until the cells on the wall of the culture flask were completely detached. The cells on the wall of the culture flask were completely suspended into the culture medium until it became a single-cell suspension. The cells were passaged at a ratio of 1:2–3. The cells were frozen after 2–3 generations of passaging. Cells at the exponential growth stage were harvested for further analysis.

#### 2.6.2. Cell Grouping

1. Control group: DMEM containing 4% FBS was used for cell culture.
2. Steroid group: DMEM containing 4% FBS and 100 μMDEX was used for cell culture.
3. Different concentrations of proanthocyanidins group: MG63 cells were cultured with DMEM (4% FBS and 100 μMDEX) containing different concentrations of PACs prepared in advance and divided into 10 μM/mL group, 20 μM/mL group, and 40 μM/mL group according to different concentrations of PACs.

### 2.7. Cell Apoptosis Assay

Annexin V-FITC apoptosis detection kit (Key-based biology, Nanjing, Jiang Su, China) was used to detect the apoptosis rate of MG-63 cells. MG-63 cells at the exponential growth stage with good growth condition were cultured in 6-well plates (5 × 10^5^ cells/well) at 37 °C and 5% CO_2_ for 24 h. Cells were treated according to grouping. After trypsin digestion, the cells were rinsed twice with PBS and centrifuged for 5 min at 1200 rpm. The cells were added with 500 μL binding buffer, resuspended, and mixed with 5 μL Annexin V-FITC followed by 5 μL propidium iodide (PI). After mixing, the cells were evaluated by flow cytometry (Beckman, Pasadena, CA, USA).

### 2.8. Western Blot Analyses

MG-63 cells at the exponential growth stage were inoculated in 25 cm^2^ cell culture flasks, and when the cells reached 80% confluence, drug was administered and grouped according to Section 2.6.2. The culture was continued for 48 h, and then 300 μL of lysis solution was added to each flask (adjusted according to the number of cells), and the cells were lysed on ice for 30 min, collected at 4 °C, 12,000 r·min^−1^, and centrifuged for 30 min (centrifuge radius 10 cm). The cell supernatant was taken for Bicinchoninic Acid Assay (BCA) protein concentration determination. The protein samples were separated through sodium dodecyl sulfate-polyacrylamide gel electrophoresis (SDS-PAGE), transferred onto polyvinylidenefluoride (PVDF) membrane, and 5% skimmed milk was closed for 1 h at room temperature (the closing time was extended appropriately according to the background of the bands). After washing four times with Tris Buffered Saline with Tween (TBST) for 5 min/time, the membrane was added with PI3K, p-PI3K, AKT, p-AKT, Bcl-xL, BCL2-Associated X (BAX), Caspase3, and cleaved-Caspase3 antibodies (dilution ratio of 1:1000) and incubated overnight at 4 °C. Four washes were given using TBST, 5 min/wash, HRP horseradish enzyme-labeled goat anti-rabbit antibody (1:5000) was added, and it was then incubated for 1 h in a shaking bed. Four washes of TBST were given, 5 min each wash, ECL luminescence reagent was added for development, and a multifunctional molecular imaging system was used for image collection. β-actin was employed as the internal reference, and ImageJ was employed for grayscale value analysis of the protein bands.

### 2.9. Statistical Analysis

SPSS analysis was performed with IBM SPSS Statistics for Windows, v25.0. The data of each experiment were expressed as mean ± standard deviation (x ± s). One-way ANOVA was utilized for the comparisons among multiple groups, and independent samples *t*-test was applied for the comparisons between two groups.

## 3. Results

### 3.1. Proanthocyanidins Treat Early DEX-Induced Bone Loss by Inhibiting Osteoblast Apoptosis In Vivo

**Figure 1:**
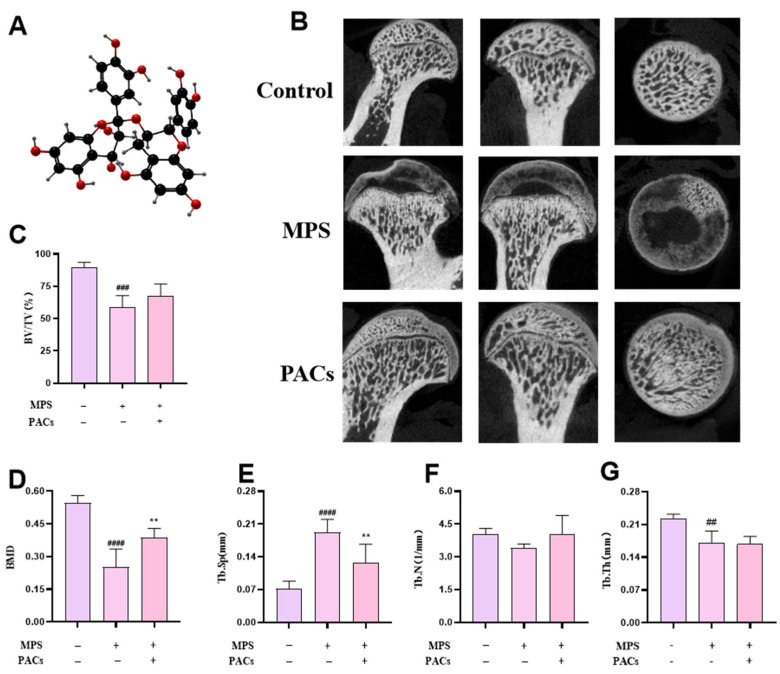
PACs inhibit in vivo bone loss in SONFH. (**A**) The molecular formula of PACs. (**B**) Representative Micro-CT scans of the femoral heads of control, MPS, and PACs groups.(**C**–**G**) Quantitative analysis of BV/TV, BMD, Tb.Sp, Tb.N, and Tb.Th. Data are presented as *t* mean ± SD, *n* = 5. ^##^ *p* ≤ 0.01, ^###^ *p* < 0.001, ^####^ *p* ≤ 0.0001 versus the control group; ** *p* < 0.01 versus the MPS group. Abbreviations: PACs: proanthocyanidins; MPS: methylprednisolone; BV/TV: bone volume/tissue volume; BMD: bone mineral density; Tb.N: trabecular number (1/mm); Tb.Th,: trabecular thickness (mm); TB.Sp: trabecular separation (mm).

To investigate the effect on MPS-induced SONFH, we treated a DEX-induced SONFH rat model with PACs and analyzed it using micro-CT and H&E staining for morphological observations. Micro-CT images and parameters demonstrated that the rats in the MPS group had sparse bone trabeculae and significant bone loss at the femoral head area that appeared as an irregular hypodense shadow, compared with the control rats (Figure 1B). Moreover, BV/TV, TB.Th, and TB.N were reduced, and TB.SP was elevated (Figure 1C–F). In contrast, the morphology of the femoral head was significantly better in the PACs group than in the steroid group, and the micro-CT parameters BV/TV, TB. Th, and TB. N were increased and the TB. SP levels were lower in the PACs group than in the steroid group (Figure 1B–F).

**Figure 2:**
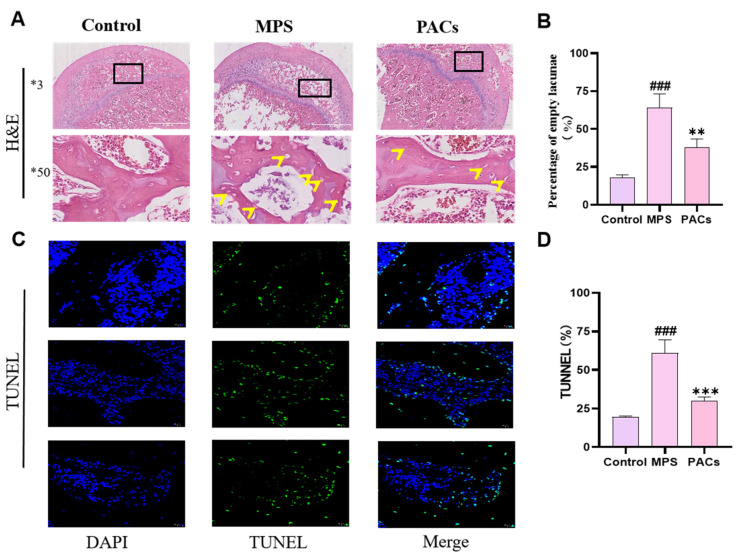
Assessment of osteonecrosis and apoptosis in the rat models. (**A**) H&E staining of femoral head in the control, MPS, and PACs groups. Empty lacunae are indicated by yellow arrows. (**B**) Quantitative evaluation of the percentage of empty lacunae (%). (**C**) TUNEL staining was performed in the control, MPS, and PACs groups. (**D**) The number of total and positive cells in each image were counted to calculate the apoptosis rate (%). *3 represents slices observed at 3x, *50 represents slices observed at 50x ^###^ *p* < 0.001 versus the control group, ** *p* < 0.01, *** *p* < 0.001 versus the MPS group. Abbreviations: PACs: proanthocyanidins; MPS: methylprednisolone; H&E: hematoxylin and eosin; TUNEL: TdT-mediated dUTP nick end labeling.

The H&E staining results of the femoral head tissue sections indicated that there was no obvious necrosis in the control group, and compared with the control group, the rats in the MPS group had sparse internal bone trabeculae, a higher rate of empty bone traps, and obvious necrosis in the femoral head. In contrast, the rats in the PACs group did not show obvious necrosis in the bone tissue, had fewer bone trabeculae replaced by necrotic tissue, and had a significantly lower rate of empty bone traps. The results of H&E staining were the same as those of Mirco-CT, indicating PACs had a protective effect against the loss of bone trabeculae in MPS-induced femoral head necrosis (Figure 2A,B). Decalcification will cause certain empty bone traps, but the rate is low; generally, normal bone tissue decalcification caused by empty bone traps does not exceed 10%, whereas the rate of empty bone traps in necrotic tissue can reach 40–80% [24].

Femoral head tissues were also examined for apoptosis using TUNEL staining, and the finding demonstrated that the control group had the least number of positive cells. The number of apoptotic cells was remarkably higher in the MPS group than in the control group, whereas the number of positive cells was markedly lower in the PACs group than in the MPS group. The consistent results suggest that PACs can reduce MPS-induced apoptosis in rat femoral head cells (Figure 2C,D).

### 3.2. Network Pharmacological Analysis of Proanthocyanidins and Steroid-Induced Femoral Head Necrosis

**Figure 3:**
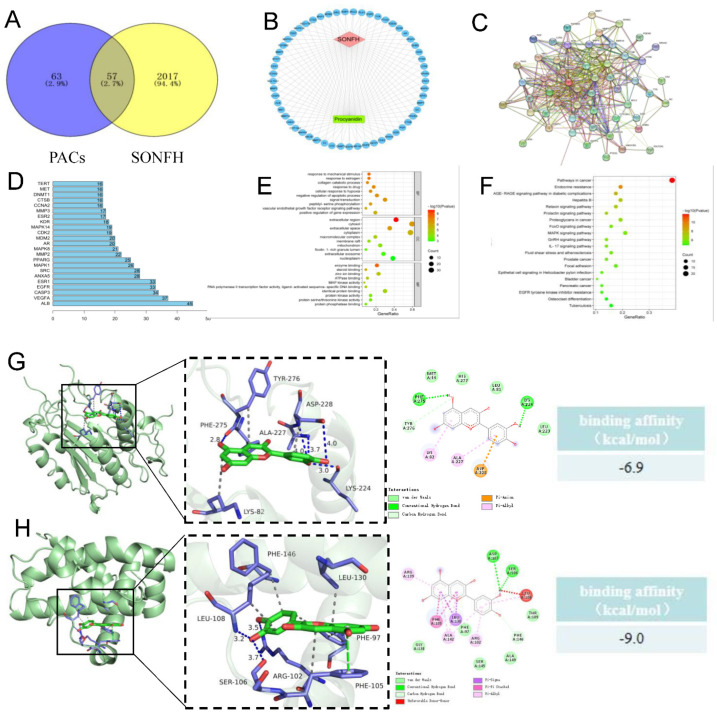
Network pharmacology analysis predicted that targets of PACs may interact with targets of SONFH. (**A**) Venn diagram displaying the common and unique targets of drug and disease. There were 120 drug targets, 2074 disease targets and 57 common drug-disease targets. (**B**) The common drug-disease targets were imported into Cytoscape software to build a disease-target-component network, in which green nodes represent PACs, blue nodes represent common targets, and red nodes represent disease. (**C**) The common drug-disease targets were retrieved in the STRING database to construct a protein–protein interaction network. (**D**) Topological analysis was performed to draw a bar graph of the core target using R 4.2.1. (**E**) The biological process, cell component, and molecular function were obtained by GO enrichment analysis of DAVID database. (**F**) KEGG enrichment analysis was conducted in DAVID database, and the top 20 significant terms were visualized by bubble chart. (**G**) The molecular docking results indicated that PACs could spontaneously and stably bind to the cavity of the Caspase3 and interact with the surrounding amino acids; the binding energy of this molecular docking is −6.9 kcal/mol. (**H**) The molecular docking results indicated that PACs could spontaneously and stably bind to the cavity of the Bcl-xL and interact with the surrounding amino acids; the binding energy of this molecular docking is −9 kcal/mol. Abbreviations: PACs: proanthocyanidins; SONFH: steroid-induced osteonecrosis of the femoral head; DAVID: The Database for Annotation, Visualization and Integrated Discovery; Search Tool for the Retrieval of Interaction Gene/Proteins; GO: Gene Ontology; KEGG: Kyoto Encyclopedia of Genes and Genomes; Bcl-xL: Recombinant Human B-Cell Leukemia/Lymphoma 2 XL.

The structures of the proanthocyanidins were imported into three databases, and 120 targets were obtained by correcting and unifying the target names in UniProt database and deduplicating the targets. By searching the OMIM and Genecards databases, a total of 2074 disease targets were obtained after de-duplication. We entered 120 drug targets and 2074 disease targets on the Venny 2.1 online software mapping tool platform, drew a Venn diagram, and obtained 57 common drug-disease targets after taking the intersection of the two (Figure 3A). PACs and 57 common drug-disease targets were imported into Cytoscape software to create a “disease-target-component” network diagram (see Figure 3B). Green, blue, and red represent PACs, the 57 common targets, and diseases, respectively. The above drug-disease common targets were inputted into the STRING database for searching, the protein type and minimum interaction threshold were set as “Homo sapiens” and 0.4, respectively, and the isolated nodes were removed to build the PPI network (Figure 3C). The size and color shades of the nodes change according to the size of the node degree value. The PPI network was visualized by Cytoscape 3.9.2. Topological analysis was conducted using the Network Analyzer tool, and the genes with degree values above the mean score were selected as core targets by degree-ranking. The targets were plotted as bar graphs using R 4.2.1 (Figure 3D). The 57 common targets were enriched using GO analysis in the David database to obtain three parts: biological processes, cellular components, and molecular function. GO date indicated that the intersecting genes were enriched in 247 biological process pathways, 37 cellular component expression processes, and 56 molecular function-related processes. The top 10 *p*-value ranked pathways in each part were selected and plotted on a bar graph bubble diagram (Figure 3E). A total of 98 KEGG pathways were obtained after KEGG enrichment analysis of 57 common targets in the DAVID database, and the top 20 pathways were chosen by *p*-value ranking for KEGG enrichment (Figure 3F). Visualization analysis was performed after molecular docking, and AutoDock-Vina was employed for semi-flexible docking. The scores and interaction energy results are presented in the table. According to the literature [25], an autoDock-vina docking score below −7.0 signifies a high affinity between the target and the compound, whereas a score between −5.0 and −7.0 suggests a moderate binding ability of the small molecules with the target (Figure 3G,H).

### 3.3. Proanthocyanidins Promote the Proliferation of MG-63 Human Osteogenic Sarcoma Cells and Inhibit DEX-Induced Apoptosis 

**Figure 4:**
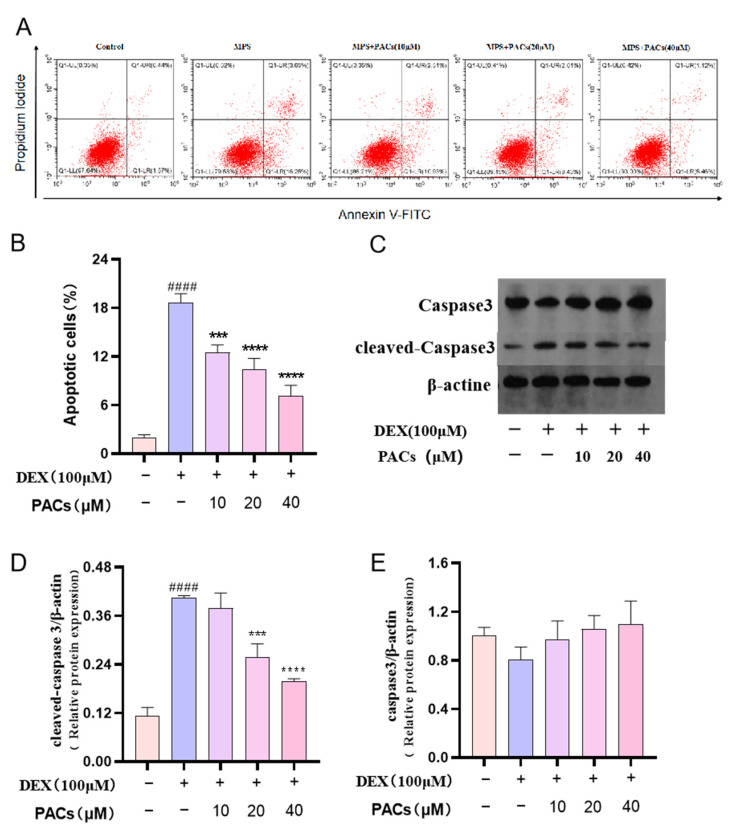
Evaluation of the effect of PACs at 10 μM, 20 μM, and 40 μM concentrations on Dex-induced apoptosis of MG-63 cells. (**A**) The apoptosis rate of MG-63 cells treated with Dex was determined by flow cytometry. (**B**) Quantitative evaluation of the MG-63 apoptotic cells. (**C**) The expression of caspase3 and cleaved caspase3 in MG-63 cells treated with Dex and PACs (10 μM, 20 μM, and 40 μM) were detected by Western blotting. (**D**,**E**) Quantitative evaluation of the protein levels of caspase 3 and cleaved caspase 3 after different treatments. ^####^ *p* < 0.0001 compared with the control group; *** *p* < 0.001, **** *p* < 0.0001 versus the MPS group. Abbreviations: PACs: proanthocyanidins; Dex: dexmedetomidine; MG-63 cells: human osteoblast-like sarcoma cell line; PI: Propidium iodide.

The apoptosis analysis revealed that DEX significantly increased the apoptosis of MG-63 cells compared to the control group. However, different doses of PACs attenuated the DEX-induced apoptosis in MG-63 cells (Figure 4A,B). Expression of the apoptotic protein Caspase-3 was examined using Western blotting. Cleaved Caspase-3 expression decreased significantly when PACs reached 20 μM (Figure 4C–E). This indicated that different doses of PACs promoted osteoblast resistance to DEX-induced apoptosis. 

### 3.4. Proanthocyanidins Rescue DEX-Induced Apoptosis in Osteoblasts via the PI3KAKT Axis

**Figure 5:**
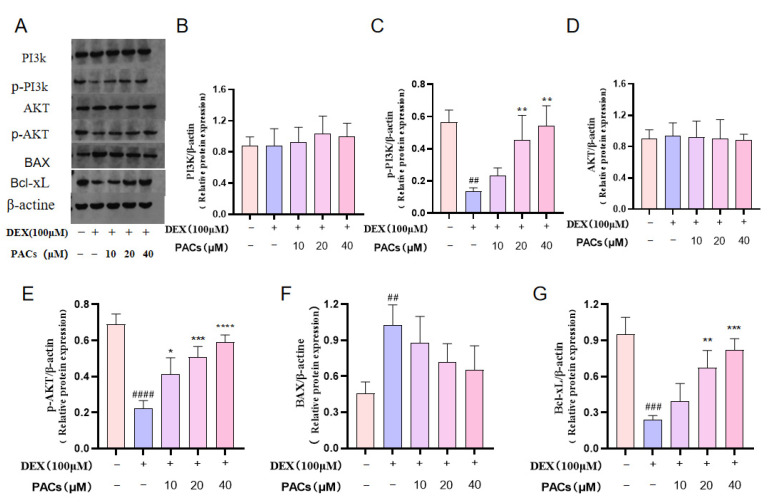
Expression of PACs in PI3k/AKT axis in DEX-treated MG-63 cells. (**A**) Western blotting was performed to detect the expression levels of PI3K, p-PI3K, AKT, p-AKT, BAX, and Bcl-xL in MG-63 cells in DEX-treated MG-63 cells treatment with DEX (100 μM,48 h) and different PACs concentrations (10 μM, 20 μM, and 40 μM). (**B**–**G**) Quantitative evaluation of PI3K, P-PI3K, AKT, p-AKT, BAX, and Bcl-xL. ^##^ *p* < 0.01, ^###^ *p* < 0.001, ^####^ *p* < 0.0001 versus the control group; * *p* < 0.05, ** *p* < 0.01, *** *p* < 0.001, **** *p* < 0.0001 versus the MPS group. Abbreviations: PACs proanthocyanidins; DEX: dexmedetomidine. PI3K: Phosphoinositide 3-kinase; p-PI3K: phosphorylate Phosphoinositide 3-kinase; AKT: protein kinase B; p-AKT: phosphorylate protein kinase B; BAX: BCL2-Associated X; Bcl-xL: Recombinant Human B-Cell Leukemia/Lymphoma 2 XL.

To explore the mechanism of action of PACs in the DEX-induced apoptosis of osteoblasts, Western blotting was performed to determine the expression levels of the PI3K-AKT axis-related proteins. The results showed that high doses of DEX significantly activated BAX expression, which in turn cleaved Caspase-3 and led to apoptosis. When PACs were added to the MG-63 medium containing DEX, the protein levels of BAX and Bcl-xL were decreased and increased, respectively (Figure 5A–G). This indicated that the anti-apoptotic effect of PACs was mainly achieved by regulating the AKT/Bcl-xL signaling pathway.

## 4. Discussion

PACs refer to a diverse group of natural compounds that contain one or more phenol rings and are commonly found in plant sources; their common feature is that they can all produce anthocyanins when heated in acidic media [26]. PACs are pigment components that are widely found in various plants and are powerful antioxidants that have a role in strengthening bones [27]. Previously, it was found that proanthocyanidins have an inhibitory effect on apoptosis [28], and some studies show the effects of PACs on femoral head necrosis. PACs are believed to have a potential therapeutic effect against femoral head necrosis, but the exact mechanism remains unclear [29].

We first performed an in vitro study in rats, which was consistent with the findings of previous literature on a rabbit model of femoral head necrosis [10]. Therefore, we constructed a steroid-induced femoral head necrosis model in SD rats [30] while administering procyanidin gavage treatment. After seven weeks of treatment, the mice were anesthetized with an excessive dose of 10% sodium pentobarbital, and the treatment effect was determined by performing micro-CT scans, H&E staining, and TUNEL staining of the femoral head bilaterally. The H&E staining and micro-CT analysis revealed that the rate of hollow bone depression in the femoral head of the hormone group was significantly increased compared to the control group. TUNEL staining demonstrated that the number of positive cells in the hormone group was remarkably increased, indicating that morphological changes in the femoral head occurred under excessive steroid induction. The blood supply to the femoral head was damaged, cells were over-apoptotic, and symptoms of early necrosis appeared. After treatment with proanthocyanidin, H&E staining and micro-CT showed that the hollow bone lacunation rate of the femoral head was markedly reduced compared to the hormone group, and TUNEL staining indicated that the number of positive cells in the PACs group was markedly reduced compared to the hormone group, which was consistent with the findings of Chen et al. [31]. This suggests that PACs have a protective effect against SONFH induced by excessive steroids, and could inhibit steroid-induced apoptosis.

To further clarify the mechanism of PACs in the treatment of SONFH, we adopted a network pharmacology and bioinformatics approach to construct a multi-level network of “disease-phenotype-gene-drug,” as a regulatory mechanism in the body is usually not governed by a single signaling pathway, but a unified and complex regulatory network. Different signaling pathways and targets have a certain degree of signal transfer to each other; therefore, the active ingredients of drugs are not only directly combined with the targets of the ingredients, but also combined directly and indirectly. Through the construction and mining of the PPI data, 57 proanthocyanidins associated with femoral head necrosis were identified. Combining GO and KEGG enrichmentanalyses, although the “pathway in cancer” was classified as the first KEGG pathway in the current study, when all differentially expressed gene (DEGs) classified under this pathway were re-analyzed, most of the genes were also classified as apoptotic genes, and most of the top-ranked pathways were also strongly associated with apoptosis [32]. Therefore, it can be said that PACs exert their therapeutic effects on femoral head necrosis mainly through anti-apoptosis, promoting bone regeneration and vascular regeneration. Among the core targets predicted based on topological analysis, caspase-3, which ranks third, plays an irreplaceable role in apoptosis, and its activation can be blocked by the B-cell lymphoma-2 (BCL-2) family [33]. In contrast, the activity of BCL-2, downstream of the PI3K/AKT signaling pathway, is influenced by AKT phosphorylation, which regulates apoptosis [34].

Femoral head necrosis was accompanied by osteoblast apoptosis and decreased bone formation [35]. In contrast, osteoblasts are the primary functional cells responsible for bone formation and synthesis, secretion, and mineralization of the bone matrix during development [36]. The inhibition of osteoblast apoptosis in bone tissue cells allows treatment at an early stage of osteonecrosis [37,38]. Previous studies demonstrated that enhanced PI3K/AKT signal transduction can induce osteoblast proliferation and delay their apoptotic time [39]. Therefore, we performed in vitro MG-63 cell culture and observed a significant increase in apoptosis after DEX treatment according to the flow cytometry results, as previously reported [40], whereas the addition of different doses of PACs after DEX treatment significantly inhibited DEX-induced apoptosis. We also determined the expression levels of PI3K, p-PI3K, AKT, and p-AKT and measured the expression of apoptosis-related proteins (e.g., Bcl-xL, BAXand Caspase3) via Western blotting. The expression levels of p-PI3K, p-AKT, and Bcl2-XL were significantly decreased in DEX-treated MG-63 cells, whereas apoptotic proteins such as Bax and Caspase3 were overexpressed, indicating that DEX has an inhibitory effect on the PI3K/AKTaxis. The expression levels of p-PI3K, p-AKT, and Bcl2-XL gradually increased after adding different doses of PACs, reaching the highest expression at 40 μM, whereas the expression of apoptotic proteins (Bax and Caspase3) was inhibited, indicating that PACs could reverse the inhibitory effect of DEX on PI3K/AKTsignal transduction. According to network pharmacological analysis, PI3K/AKT is an upstream apoptotic pathway. PACs can depolymerize and free Bcl-xL, playing an anti-apoptotic role. Activated Akt directly catalyzes the phosphorylation of caspase-3, inactivates Caspase-3-induced apoptosis, and inhibits the apoptosis of osteoblasts induced by steroid-induced femoral head necrosis [41,42,43].

There are some limitations in this study. First, network pharmacology can only predict the relevant pathways with limited accuracy. Second, only osteoblast apoptosis was experimentally verified. We will continue to investigate the pre-use of PI3K inhibitors or its downstream targets to further confirm the role of PACs in regulating the PI3K/AKT axis. Further studies on other important cells or the related pathways of PACs during osteonecrosis are still warranted.

## 5. Conclusions

In vivo animal studies in rats showed that proanthocyanidins have a protective effect against the bone loss caused by steroid-induced femoral head necrosis; the PI3K/AKT signaling pathway as an upstream pathway of apoptosis was shown to be correlated with the inhibition of osteoblast apoptosis by PACs based on a network pharmacology approach. In vitro osteoblast experiments clearly showed that PACs can inhibit osteoblast apoptosis through the PI3K/AKT and promote cellular value addition. Our study suggests a potential therapeutic application of PACs in femoral head necrosis, and its mechanism of action may be related to the regulation of the PI3K/AKT/Bcl-xL axis to inhibit osteoblast apoptosis.

## Data Availability

The authors confirm that all data underlying the findings are fully available and can be obtained after submitting a request to the corresponding author.
